# The balance model of honest sexual signaling

**DOI:** 10.1111/evo.14436

**Published:** 2022-02-01

**Authors:** Lutz Fromhage, Jonathan M. Henshaw

**Affiliations:** ^1^ Department of Biological and Environmental Science University of Jyvaskyla Jyvaskyla 40014 Finland; ^2^ Institute of Biology I (Zoology) University of Freiburg Freiburg 79104 Germany

**Keywords:** Models/simulations, selection—sexual, signaling/courtship, trade‐off

## Abstract

Costly signaling theory is based on the idea that individuals may signal their quality to potential mates and that the signal's costliness plays a crucial role in maintaining information content (“honesty”) over evolutionary time. Although costly signals have traditionally been described as “handicaps,” here we present mathematical results that motivate an alternative interpretation. We show that under broad conditions, the multiplicative nature of fitness selects for roughly balanced investments in mating success and viability, thereby generating a positive correlation between signal size and quality. This balancing tendency occurs because selection for increased investment in a fitness component diminishes with the absolute level of investment in that component, such that excessively biased investments are penalized. The resulting interpretation of costly signals as balanced (albeit not necessarily equal) investments may be a widely applicable alternative to the traditional “handicap” metaphor, which has been criticized for its non‐Darwinian connotation of selection for “waste” rather than efficiency. We predict that accelerating returns on viability are necessary to undermine honesty. This prediction depends crucially on the assumption that mating success and viability contribute multiplicatively (rather than additively) to an individual's fitness.

In many animal species, males (or, more rarely, females) exhibit conspicuous secondary sexual traits that are targets of mate choice by the opposite sex, potentially because they provide reliable information about an individual's condition, health, and/or social status (Andersson 1994; Dougherty 2021). This raises the question: what prevents poor‐quality individuals from “cheating”, that is, expressing traits that give an exaggerated impression of their actual quality? A popular answer to this question is Amotz Zahavi's handicap hypothesis (Zahavi 1975; reviewed in Penn and Számadó 2020), which states that signal traits impose survival costs that can more easily be borne by high‐quality individuals. The handicap hypothesis was initially met with skepticism (e.g., Kirkpatrick 1986), but became widely accepted after Grafen (1990) presented a theoretical model showing that honest signaling can be stable when individuals strategically choose their signal strength conditional on their quality. Although some researchers took Grafen's model as setting a new benchmark for interpreting the handicap hypothesis, Zahavi's writings remained a source of confusion. This is because Zahavi applied his handicap terminology to several distinct ideas, some of which are incompatible with Darwinian logic (Penn and Számadó 2020). In particular, Zahavi claimed that signals evolve through a special kind of selection which—in contrast to natural selection—favors waste and inefficiency (Zahavi 1981, 1987; Zahavi and Zahavi 1997). This claim reflects the obsolete view that natural selection maximizes individual survival rather than gene propagation, such that a trait that reduces survival counts as wasteful regardless of its effect on reproduction. Although Grafen's (1990) model was perfectly Darwinian, he nevertheless embraced the Zahavian rhetoric of wastefulness, stating, for example (p. 532): “The evolutionary stability of persuasive signaling necessitates honesty, which necessitates waste.” Grafen (1990) also derived a more specific result, which states that, for honesty to be stable, the marginal cost of advertising should be greater for worse males (the “decreasing‐marginal‐cost criterion”; Getty 2006). Accordingly, borrowing an example from Getty (2006, p. 84), “a high‐quality peacock with 100 eye spots on its tail should be able to add one more spot at less viability cost than could a poor‐quality peacock with 100 eye spots.” Yet, as Getty (2006) pointed out, the decreasing‐marginal‐cost criterion rests on the implicit assumption that the costs and benefits of signaling combine in an additive manner—which cannot be true if benefits arise in terms of mating success and costs arise in terms of viability. Without the unrealistic assumption of additivity, the decreasing‐marginal‐cost criterion boils down to the more general criterion that the marginal net fitness benefit of increased signal strength should increase with quality (Getty 2006): that is, a high‐quality peacock with 100 eye spots should gain more fitness from adding one more spot than could a poor‐quality peacock with 100 eye spots. Unfortunately, this criterion is so abstract that it is unclear how easily it might be satisfied (Getty 2006). Because there is no logical necessity for net fitness benefits to increase in this way, one may get the impression that honest signaling only works if the quality‐dependent fitness functions happen to have very specific shapes by coincidence (e.g., a product of exponential functions, as assumed by Iwasa et al. 1991; Johnstone et al. 2009).

By contrast, here we argue that there is a powerful reason why fitness functions should often be of the right kind to support honest sexual signaling. We locate this reason in the multiplicative nature of the mating versus viability trade‐off that is commonly supposed to underlie costly sexual signaling. To develop our argument, let us briefly step back to compare different kinds of trade‐offs in a more general setting.

## Additive and Multiplicative Trade‐Offs

A trade‐off describes a situation in which two or more desirable (i.e., fitness‐enhancing) quantities cannot be maximized simultaneously, requiring a decision that balances conflicting needs. We will focus in particular on trade‐offs where fitness can be expressed either as the sum of fitness components (“additive” trade‐offs) or as the product of fitness components (“multiplicative” trade‐offs; also see Houston et al. 2003). One way to formalize the idea that fitness components $x_{1}\left(u_{1}\right),\;x_{2}\left(u_{2}\right),\;\text{…},\;x_{n}\left(u_{n}\right)$ are subject to a trade‐off is to postulate a constraint $R=u_{1}+u_{2}+\;\cdots \;+u_{n}$ that must be met by the allocations $u_{1},\;u_{2},\;\text{…},\;u_{n}$ to these components (with $u_{i}\geq 0$). The simplest interpretation of this is that *R* represents the total amount of some limiting resource (e.g., energy: Somjee 2021) accumulated by the organism before the allocation decision. More generally, *R* can be envisaged as an abstract measure of “quality” that constrains the multivariate phenotypic space that an individual can access via its developmental and behavioral decisions. Hence, *R* may also capture an organism's capacity to acquire resources and to use them efficiently to express adaptive traits. Variation in *R* may thus reflect both environmental (e.g., microhabitat experienced during development) and genetic factors (e.g., load of deleterious alleles; pathogen resistance; possession of locally beneficial alleles).

Let us write fitness as a function $W(x_{1},\;x_{2},\;\text{…},\;x_{n})$ of the fitness components *x_i_*. Suppose that an optimal strategy for a given individual involves a nonzero investment in the fitness components *x_i_* and *x_j_* (we will consider the case of zero investment in a fitness component below). It follows that the marginal fitness increase from investing in each component must be equal at this optimum—otherwise it would pay to reallocate resources toward the component with the highest marginal fitness increase (the “marginal advantage theorem”; Lloyd 1988). Formally, this means that for any fitness components *x_i_* and *x_j_* with $u_{i},u_{j}>0$ at the optimum, we must have

(1) $${\left.\frac{\partial W}{\partial u_{i}}\right|}_{u_{i}=u_{i}^{*}}={\left.\frac{\partial W}{\partial u_{j}}\right|}_{u_{j}=u_{j}^{*}}.$$

Note that these derivatives are evaluated at the optimum values of *u_i_* and *u_j_*, respectively, denoted here as $u_{i}^{*}$ and $u_{j}^{*}$. What this implies about optimal resource allocation depends on the functional form of *W*, which reflects the type of trade‐off at hand. If the fitness components can be measured in the same currency (e.g., the number of offspring produced, assuming offspring have equal reproductive value), fitness can be expressed as

(2) $$W\;\left(x_{1},\;x_{2},\;\text{…},\;x_{n}\right)=x_{1}+x_{2}+\;\cdots \;+x_{n}.$$

For example, consider the trade‐off between producing intra‐ versus extrapair offspring. Here, the fitness value of a given offspring to its father may be independent of how many offspring of the other type he sires. Similar considerations may also apply to production of sons versus daughters, to production through male versus female function (in hermaphrodites), to production of offspring versus other relatives (in cooperative breeders), or various combinations of these possibilities.

Substituting equation (2) into equation (1), we learn that if fitness is additive and an optimal strategy involves nonzero investments in both *x_i_* and *x_j_*, then

(3) $${\left.\frac{dx_{i}}{du_{i}}\right|}_{u_{i}=u_{i}^{*}}={\left.\frac{dx_{j}}{du_{j}}\right|}_{u_{j}=u_{j}^{*}}.$$

In other words, at the optimal allocation, the marginal absolute increase in each fitness component with respect to investment must be equal.

Alternatively, a trade‐off may exist between multiplicative fitness components, that is, those whose product is fitness, as

(4) $$W\;\left(x_{1},\;x_{2},\;\text{…},\;x_{n}\right)=x_{1}\;x_{2}\;\cdots \;x_{n}.$$

For example, male fitness might be expressed as: (expected life span) * (proportion of life spent reproductively active) * (mating rate during this time) * (number of fertilizable eggs per mating) * (expected paternity share) * (recruitment probability per offspring) * (reproductive value per offspring recruited). Substituting equation (4) into equation (1) yields

(5) $${\left.\frac{dx_{i}}{du_{i}}\right|}_{u_{i}=u_{i}^{*}}\;\prod_{k\neq i}x_{k}\left(u_{k}^{*}\right)={\left.\frac{dx_{j}}{du_{j}}\right|}_{u_{j}=u_{j}^{*}}\;\prod_{k\neq j}x_{k}\left(u_{k}^{*}\right).$$

Thus, optimal allocation equalizes the marginal increase of each fitness component *x_i_* with respect to investment, weighted by the product of all other components. For a given pair of fitness components *x_i_* and *x_j_*, this can also be expressed as

(6) $$\frac{1}{x_{i}\left(u_{i}^{*}\right)}\;\left({\left.\frac{dx_{i}}{du_{i}}\right|}_{u_{i}=u_{i}^{*}}\right)=\frac{1}{x_{j}\left(u_{j}^{*}\right)}\;\left({\left.\frac{dx_{j}}{du_{j}}\right|}_{u_{j}=u_{j}^{*}}\right).$$

In other words, the *marginal proportional increase* (known in economics as “elasticity”) must be equal for each fitness component that receives a nonzero investment. This means that a small absolute increase of investment *u_i_* in any such fitness component (while holding other investments unchanged) must lead to the same proportional increase in that component, and hence in fitness itself. For example, increasing mating rate by 1% will increase fitness by 1%, all else being equal. Each side of equation (6) can be interpreted as a selection gradient, that is, as a proportional derivative of the fitness component with respect to investment:

(7) $$S_{i}\;\left(u_{i}\right)=\frac{{x^{\prime }}_{i}\;\left(u_{i}\right)}{x_{i}\;\left(u_{i}\right)}.$$

Hence, equation (6) says that if an optimal strategy invests in both *x_i_* and *x_j_*, then $S_{i}\left(u_{i}\right)=S_{j}\left(u_{j}\right)$ at the optimum. Similarly, if an optimal strategy invests in *x_i_* but not *x_j_* (i.e., $u_{i}>0$ but $u_{j}=0$), then $S_{i}\left(u_{i}\right)\geq S_{j}\left(0\right)$.

Interestingly, previous theory on honest signaling has sometimes combined aspects of multiplicative and additive models. For instance, Iwasa et al. (1991) and Johnstone et al. (2009) assume that fitness is given by the product of exponential components. This product becomes additive on the logarithmic scale, allowing the authors to apply mathematical techniques that were developed for the case of additive trade‐offs. In contrast, the arguments we develop below apply directly to multiplicative trade‐offs without the need to assume particular functional forms. Instead, we assume that trade‐offs arise due to reliance on a common limiting resource.

## Signal Honesty

To link these general considerations to sexual signaling, let us suppose that fitness is a multiplicative function of mating success *x*_1_ and viability *x*_2_. This might hold approximately for males in species with no paternal investment in offspring, such that a male's mating success is a good proxy for his reproductive success. Furthermore, let us assume that investment *u*_1_ is translated into mating success *x*_1_ via expressing a signal trait $t_{1}$, such that $t_{1}$ increases monotonically with *u*_1_ and *x*_1_ increases monotonically with $t_{1}$. Thus, $t_{1}$ provides reliable information about *R* whenever $u_{1}$ increases monotonically with R, allowing us to use the latter relationship as our criterion for signal honesty. For simplicity, here we focus on the case where signal investment reduces viability indirectly, by consuming resources that would otherwise be invested in viability. However, as we show in the appendix, an analogous argument applies when signals also reduce viability directly (e.g., by attracting predators).

**Figure 1:**
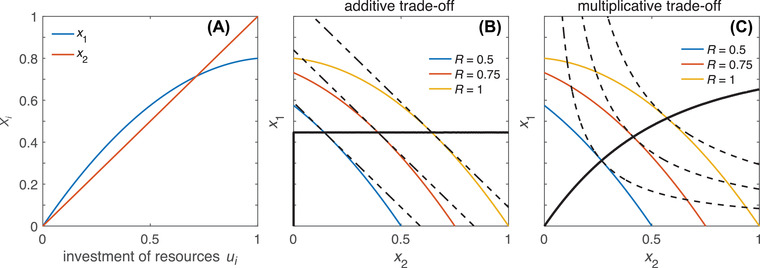
Example where a multiplicative (but not an additive) trade‐off ensures signal honesty. (A) Fitness components *x*_1_ (mating success) and *x*_2_ (viability) as functions of the absolute amount of resources allocated to them, shown here as $x_{1}=1.5u_{1}-0.7u_{1}^{2}$ and $x_{2}=u_{2}$. We assume that mating success is an increasing function of signal strength, which in turn increases with investment *u*_1_. (B) Additive trade‐off between *x*_1_ and *x*_2_, with fitness given by $W=x_{1}+x_{2}$. Colored lines represent achievable trait combinations (phenotype sets) for individuals with fixed resource levels (see legend within panel). Dashed lines are fitness isoclines that connect hypothetical trait combinations yielding the same fitness. For each colored line (resource level), the optimal allocation occurs at the point where it touches the highest fitness isocline, corresponding to the highest achievable fitness for a given resource budget. The solid black line connects optimal trait combinations that correspond (from left to right) to increasing resource budgets. The solid black line's flat slope shows that individuals of any quality have the same mating success (hence signal size); that is, there is no honest signaling of quality. This result obtains because there is only a single point (at $u_{1}=0.36$, corresponding to $x_{1}=0.45$) at which both curves shown in panel A have matching slopes, as they must at equilibrium according to equation (3). (C) Multiplicative trade‐off between *x*_1_ and *x*_2_, with fitness given by $W=x_{1}x_{2}$. Lines have the same meaning as in panel B. The solid black line's consistently positive slope indicates that signaling is honest, that is, higher quality individuals have bigger signals and hence higher mating success.

**Figure 2:**
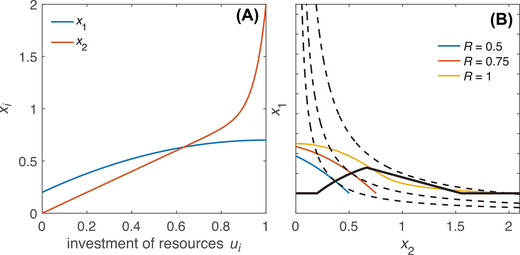
Example where a multiplicative trade‐off (i.e. fitness given by $W=x_{1}x_{2}$) fails to guarantee signal honesty. (A) Fitness components *x*_1_ (mating success) and *x*_2_ (viability) as functions of the absolute amount of resources allocated to them, shown here as $x_{1}=0.2+u_{1}-0.5u_{1}^{2}$ and $x_{2}=u_{2}+u_{2}^{20}$. We assume that mating success is an increasing function of signal strength, which in turn increases with investment *u*_1_. (B) Colored lines represent achievable trait combinations for individuals with fixed resource levels (see legend within panel). For each colored line (resource level), the optimal allocation occurs at the point where it touches the highest fitness isocline (dashed line). The solid black line connects optimal trait combinations that correspond (from left to right) to increasing resource budgets. The solid black line shows a negative slope over part of its range, indicating that higher quality individuals have smaller signals and lower mating success; that is, signaling is not honest. This outcome arises because the strongly accelerating returns of viability at high investment levels (upturned red curve on right‐hand side of panel A) induce high‐quality individuals to shift allocation toward viability at the expense of mating success.

We begin with a heuristic argument to show why honest signaling should not surprise us (for formal proofs, see the appendix). From equation (7), we can see that if we hypothetically increased *x*_2_ while its derivative $x_{2}^{\prime }$ remained constant, then *S*_2_ would decrease. This represents weaker selection for investing in *u*_2_. Now, at the optimum, we know from equation (6) that selection favors investments in *u*_1_ and *u*_2_ equally. Consequently, if the derivative $x_{2}^{\prime }\left(u_{2}\right)$ does not vary too strongly near the optimum, then there is an incentive to accompany any small increase in *u*_2_ (due to an increase in *R*) with a small (not necessarily equal) increase in *u*_1_. This selects for *u*_1_ to strictly increase with *R*, ensuring honesty, at least if the signaling strategy is constrained to be a continuous function of resource level. Even if discontinuous strategies are permitted, the optimal investment *u*_1_ is an increasing function of resources *R* whenever *S*_2_(*u*_2_) is a strictly decreasing function of investment in viability (proof in appendix). On the other hand, if a small increase in *x*_2_ is accompanied by a proportionally greater increase in $x_{2}^{\prime }$, formally, if

(8) $$\frac{{x^{\prime \prime }}_{2}\;\left(u_{2}\right)}{x^{\prime }\;\left(u_{2}\right)}>\frac{{x^{\prime }}_{2}\;\left(u_{2}\right)}{x_{2}\;\left(u_{2}\right)},$$

then *S*_2_ increases despite increasing *x*_2_. This increase in *S*_2_ creates an incentive to direct further investments to *u*_2_ rather than to *u*_1_. As a result, *u*_1_ may decrease with *R*, undermining honesty. Because both *x*_2_ and $x_{2}^{\prime }$ are assumed to be positive (i.e., *x*_2_ is a positive‐valued trait that increases with investment), condition (8) cannot be satisfied if *x*_2_ is linear ($x_{2}^{\prime \prime }=0$) or decelerating ($x_{2}^{\prime \prime }<0$). This guarantees honesty under a wide range of biologically plausible conditions, namely, those where viability does not increase with investment in an accelerating manner. As illustrated in Figure 1, the same does not hold if *x*_1_ and *x*_2_ are subject to an additive rather than a multiplicative trade‐off. Condition (8) is necessary but not sufficient for the breakdown of honesty, because the effect of a moderately accelerating function *x*_2_(*u*_2_) may be outweighed by *S*_2_’s tendency to decrease with increasing *x*_2_ (all else being equal), or by an accelerating function *x*_1_(*u*_1_). So, to undermine honesty, the acceleration of viability as a function of investment needs to be sufficiently strong (as, e.g., in Fig. 2A).

To get an intuitive sense of why the trade‐off's multiplicative nature matters, consider how the curvature of the fitness isoclines in Figure 1B, C affects their contact points with the “phenotype sets” (possible trait combinations) associated with different qualities. (This representation is inspired by fitness set theory [Levins 1962].) If phenotype sets are of similar shape (indicating that individuals of different quality face quantitatively similar trade‐offs), then high‐quality individuals reach their “target isocline” at higher values of *x*_1_ than do low‐quality individuals. That is, high‐quality individuals enjoy higher mating success than low‐quality individuals under their respective optimal strategies. By contrast, to undermine honesty, phenotype sets must differ in shape such that either (i) one of the lower phenotype sets extends upward to touch its target isocline at a relatively high value of *x*_1_ (as in Fig. 1B) or (ii) one of the higher phenotype sets extends to the right to touch its target isocline at a relatively low value of *x*_1_ (as in Fig. 2B). Such outcomes are harder to attain (i.e., require more dramatic deformations of phenotype sets) when fitness isoclines are bent in a downward‐convex manner as in Figure 1C (in contrast to Fig. 1B), making their high‐*x*_1_ and low‐*x*_1_ regions harder to reach. Even so, deviations from honesty remain possible if phenotype sets differ sufficiently strongly in shape. For example, when high values of *u*_2_ yield dramatic increases in *x*_2_ (Fig. 2A), this manifests as phenotype sets of high‐quality individuals extending strongly to the right. In other words, if enhancing viability gets cheaper toward high investment levels, then high‐quality individuals may shift their allocation to take advantage of this, even at the expense of reduced signal size (hence mating success).

**Figure 3:**
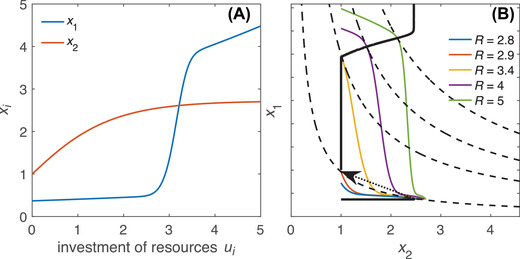
Example of honest signaling arising from a complicated allocation pattern under a multiplicative trade‐off (i.e., fitness given by $W=x_{1}x_{2}$). (A) Fitness components *x*_1_ (mating success) and *x*_2_ (viability) as functions of the absolute amount of resources allocated to them, shown here as $x_{1}=\exp\left[0.1u_{1}+\mathrm{erf}\left[3\left(u_{1}-3\right)\right]\right]$ (where erf[·] is the Gauss error function) and $x_{2}=\exp\left[1\;-\;\exp\left[-u_{2}\right]\right]$. We assume that mating success is an increasing function of signal strength, which in turn increases with investment *u*_1_. (B) Colored lines represent achievable trait combinations for individuals with fixed resource levels (see legend within panel). For each colored line (resource level), the optimal allocation occurs at the point where it touches the highest fitness isocline (dashed line). The solid black line connects optimal trait combinations that correspond (from bottom left to top right) to increasing resource budgets. At low resource levels, individuals do not invest in signaling (indicated by the flat slope at $x_{1}=0.37$, which corresponds to *u*_1_ = 0). Optimal strategies then take an abrupt turn: after a brief stretch where *x*_1_ increases while *x*_2_ remains constant (up to R≈2.9, not discernible at this resolution) follows a discontinuity, where *x*_1_ jumps upward and *x*_2_ downward (marked with an arrow). This is followed by a further period of increasing *x*_1_ with stationary *x*_2_ (up to R≈3.4), after which *x*_1_ and *x*_2_ again increase together. Despite this complexity, larger signals always imply higher quality, meaning that signaling is honest (as will always be the case when *S*_2_ is strictly decreasing: see the appendix).

We note above that if fitness is multiplicative and *S*_2_(*u*_2_) is strictly decreasing, then signaling will always be honest, in the sense that larger signals indicate higher quality (proof in the appendix). Even if this condition is met, however, the relationship between quality and investment patterns can be quite nuanced. First, if individuals gain some mating success even without signaling, then individuals with fewer resources may be best off not signaling at all (Fig. 3). In such cases, larger signals still reliably indicate higher quality, but the absence of a signal merely signifies that an individual falls into the lower range of quality. Second, investment in viability *u*_2_ may depend nonmonotonically on quality, even when signaling investment *u*_1_ is strictly increasing (Fig. 3). This can occur, for instance, when there is a sharp increase in the slope of the relationship between investment and mating success. Such changes in slope can even select for individuals to increase signal investment more than proportionally to gains in resources. This results in a discontinuity (cf. Clifton et al. 2016), where signal investment jumps upward and viability investment jumps downward (Fig. 3).

## Discussion

Here, we argue that when mating success and viability combine multiplicatively to determine fitness, then honest secondary sexual signals can evolve under broad conditions. As individual resource budgets increase, the adaptive need to keep multiplicative fitness components in balance is usually (but not always) best served by increasing investment in each. If this is the case—and assuming individuals use their optimal allocations—then an individual's resource budget (i.e., quality) can be inferred from its investment in any given fitness dimension, and in particular from signal traits that reflect investment in mating success.

The basic idea behind this reasoning can be illustrated with the following metaphor. Consider an old mechanical balance that is partly hidden from view, such that one can only see the weight carried by one of its arms. The partial view then gives full information about the total weight carried, provided we have reason to believe that the arms are, in fact, balanced. (To avoid the unwanted impression that “balanced” necessarily means “equal” here, we may allow that the balance has arms of different [known] lengths.)

In the context of sexual signaling, the reason for expecting balanced investments is rooted in the Darwinian postulate that natural selection is an optimizing process, which (in the long run) tends to shape organisms toward phenotypes that maximize their individual (inclusive) fitness (e.g., Fromhage and Jennions 2019). If fitness is multiplicative, maximizing an organism's fitness is analogous to maximizing the area of a rectangle, where the rectangle's length and width correspond to fitness dimensions in which an individual may invest. If the resource costs of increasing length or width by the same absolute amount are equal, then the most efficient strategy is to increase investment in whichever dimension is currently smallest. Even if length and width differ in cost, optimal strategies must roughly balance investments in each dimension.

Its adaptationist foundation sets our model apart from the classic work by Van Noordvijk and de Jong (1986), which showed that positive covariance between life‐history traits readily arises when individual resource budgets exhibit high variance relative to variance in allocation. Their finding has been called the “big house, big car effect” (Reznick et al. 2000), based on an analogy in economics: “if the budget is fixed, people spending more on housing should spend less on cars. In fact, the amount of expendable income is variable, and in many situations positive correlations are observed between the per‐family expenses on housing and cars” (van Noordwijk and de Jong 1986, p. 141). Yet this analogy, such as Van Noordvijk and de Jong's model, leaves open why resources should be allocated in this way. Their model simply assumes that resource acquisition is independent of relative allocation, thereby excluding the possibility that individuals adjust their allocation to maximize fitness given their budget. By contrast, our present model provides an adaptive rationale for the “big house, big car effect” as it pertains to sexual signaling and viability.

Although Grafen (1990) interpreted individual quality as an abstract property with no explicit mechanistic link to trait expression, here we have interpreted quality as a measure of an individual's resource budget. This formulation accommodates the plausible view that, say, producing an additional eye spot costs the same absolute amount of resources for peacocks of any quality, while amounting to a higher proportional investment for low‐quality peacocks. From this natural link between absolute and proportional investments arises a dependence between individual quality and signaling costs, which—without ad hoc assumptions about its shape—justifies the intuitive notion that greater resource budgets will usually lead to greater optimal spending on sexual signals.

Let us now turn to the exceptions from the prediction that higher quality individuals should produce stronger signals. We may ask: how plausible is it that viability is an accelerating function of investment, to the extent that high‐quality individuals are incentivized to neglect investment in mating success? The answer potentially depends on which aspect of viability we consider. For viability traits that are conceived of as probabilities (e.g., survival until the onset of reproduction), a diminishing rather than accelerating return on investment seems likely, at least near the theoretical maximum of 1. Biologically, we may expect that, if some causes of mortality are harder to avoid then others, then avoiding the easy ones should take priority. Thus, as an individual increases its investment in survival, each further increment may be harder to attain (i.e., require more investment) than the previous one. On the other hand, for the reproductive life span in iteroparous species—another important aspect of viability—the lack of a theoretical upper limit gives less reason to argue against accelerating returns a priori. It would therefore be desirable to study such relationships empirically, ideally by measuring or manipulating investment in survival and recording associated changes in life span.

For simplicity, we have focused on fitness trade‐offs from the perspective of the signaler, while ignoring the evolution of receiver preferences. Preferences for larger signals could conceivably be favored if quality (i.e., resource level) is partly heritable, because both male and female offspring would benefit from higher resource availability (i.e., a “good genes” model: Iwasa et al. 1991; Kuijper et al. 2012; Dhole et al. 2018). Moreover, the broad conditions for honest signaling in our model suggest that honesty should be robust to substantial variation in receiver behavior, as long as there remains a positive correlation between signal size and mating success. We consequently predict that the insights of our model will hold up in coevolutionary models of signal and preference.

A further limitation of our model is that, for simplicity, it does not allow for individuals to adjust allocation repeatedly during their lifetime, for example, in response to parasitism or other adverse life events. Yet such flexible adjustment appears to operate in several empirically documented cases of “dishonest” sexual signaling. For example, parasitized *Drosophila* males increase their courtship activity (Polak and Starmer 1998) and food‐deprived stickleback males exhibit brighter nuptial coloration (Candolin 1999), presumably in a “terminal effort” to maximize reproduction in what little time they have left. Such males then no longer face the mating‐viability trade‐off as modeled here, as their fitness gains in the short term (which is all that counts for them) may require little or no investment in viability.

Signal traits that are deemed honest and costly are often loosely labeled as handicaps without worrying about the term's history and connotations. Yet appeals have been made to usher the handicap hypothesis and its associated terminology into an “honorable retirement” (Getty 2006; Penn and Számadó 2020). There are two main arguments for this. First, the handicap hypothesis's central prediction (that costs are necessary to explain the evolution of signal reliability) has failed the test of time (Hurd 1995; Getty 1998; Számadó 1999; Lachmann et al. 2001; Higham 2013; Penn and Számadó 2020). In particular, honest signaling does not strictly require realized costs at equilibrium (as in our model); rather, it suffices that dishonesty is costly. Second, the handicap metaphor may owe much of its popularity to Zahavi's paradoxical sounding and indeed misleading rhetoric about selection for waste rather than efficiency (Zahavi 1981, 1987; Zahavi and Zahavi 1997). According to Penn and Számadó (2020, p 12), in the 1980s “Finding a model that supports the Handicap Principle became a theoretical challenge in evolutionary biology comparable to constructing a perpetual motion machine…”; that is, highly coveted yet deemed impossible. This climate generated much attention when Grafen (1990) claimed to have vindicated Zahavi (1975), thus making Zahavi's paper (and subsequent book) citation classics of the biological and social sciences in spite of their questionable aspects. From a modern standpoint, the “waste” metaphor appears to offer little of value. At the level of individual organisms, an optimal strategy might trade off one fitness component against another, but this could hardly be called “waste” if the result it to maximize fitness. On the other hand, competitive traits may very well reduce population fitness; however, this is a very general phenomenon not limited to sexual signaling (e.g., the height of trees can also be viewed in this light). Whether we can move beyond this potential source of confusion may depend, in part, on biologists’ willingness to abandon a memorable metaphor. To aid this process, here we have advocated an alternative metaphor: of costly signals as balanced investments rather than handicaps.

## CONFLICT OF INTEREST

The authors declare no conflict of interest.

## AUTHOR CONTRIBUTIONS

L.F. had the idea and wrote the first draft. J.M.H. wrote the Appendix and contributed through discussion of ideas and writing.

1

Associate Editor: T. Connallon

Handling Editor: A. McAdam

## Proof that signaling is honest

Here, we characterize the relationship between an individual's signal size (a strictly increasing function of its investment *u*_1_) and its resource level *R*. We assume throughout that the selection gradient *S*_2_(*u*_2_) on investment in viability is a strictly decreasing function of the current investment level *u*_2_. Note that

(A1)
$$S_{2}^{\prime }\;\left(u_{2}\right)=\frac{{x^{\prime \prime }}_{2}\;\left(u_{2}\right)}{x_{2}\;\left(u_{2}\right)}-S_{2}{\left(u_{2}\right)}^{2}.$$

The function *S*_2_(*u*_2_) is strictly decreasing if $S_{2}^{\prime }\left(u_{2}\right)<0$. A sufficient (but not necessary) condition is that *x*_2_(*u*_2_) is concave (i.e. $x_{2}^{\prime \prime }\left(u_{2}\right)\leq 0$).

We will prove the following theorem:

(**Honest signaling theorem**): If *S*_2_(*u*_2_) is strictly decreasing, then there is a threshold resource level *R* ^*^ such that the optimal investment in signaling is

(i) *zero for all R < R* ^*^, and
(ii) *a strictly increasing function of R for all R > R^*^*.

The cases *R* ^*^ = 0 and *R* ^*^ = ∞ are not excluded. If *R* ^*^ = 0, then optimal signaling investment always increases reliably with the resource level (i.e., an individual's signal size gives complete information about their resource level; this is sometimes known as a “separating equilibrium”). If *R* ^*^ = ∞, then individuals do not signal. For $0<R^{*}<\infty$, individuals with fewer resources do not signal, whereas those with greater resources increase their signal size along with their resource level. We will first prove the above theorem and then consider some factors influencing *R* ^*^.

We note that for a given resource level *R*, there may be more than one optimal strategy $u_{1}^{*}\left(R\right)$. The statement that optimal investment is strictly increasing should consequently be interpreted as follows: If $R_{1}<R_{2}$, then for all optimal strategies $u_{1}^{*}\left(R_{1}\right)$ and $u_{1}^{*}\left(R_{2}\right)$, we have $u_{1}^{*}\left(R_{1}\right)<u_{1}^{*}\left(R_{2}\right)$.

## Optimal investment is a nondecreasing function of resource level

We first show that optimal investment in signaling is a nondecreasing function of the resource level *R*. It is helpful to first prove a lemma about the gains in viability with increasing investment. Suppose Δ > 0 is a fixed constant and let *f*_Δ_(*u_B_*) be the proportional gain in viability when increasing investment *u*_2_ from a baseline of *u_B_* to a new level of *u_B_* + Δ:

(A2)
$$f_{\Delta }\;\left(u_{B}\right)=\frac{x_{2}\;\left(u_{B}+\Delta \right)}{x_{2}\;\left(u_{B}\right)}.$$

Note that

(A3)
$$\begin{array}{ccc} & & {f^{\prime }}_{\Delta }\left(u_{B}\right)=\frac{{x^{\prime }}_{2}\left(u_{B}+\Delta \right)}{x_{2}\left(u_{B}\right)}-\frac{x_{2}\left(u_{B}+\Delta \right){x^{\prime }}_{2}\left(u_{B}\right)}{x_{2}{\left(u_{B}\right)}^{2}}\\ & & =\;\frac{x_{2}\left(u_{B}+\Delta \right)}{x_{2}\left(u_{B}\right)}\left(S_{2}\left(u_{B}+\Delta \right)-S_{2}\left(u_{B}\right)\right)\;. \end{array}$$

Because *S*_2_ is strictly decreasing and Δ > 0, we have $f_{\Delta }^{\prime }\left(u_{B}\right)<0$. Hence, *f*_Δ_ is also a strictly decreasing function. This means that the proportional increase in viability when increasing investment by a fixed absolute amount Δ is a strictly decreasing function of baseline investment *u_B_*.

Now, suppose there exist $R_{1}<R_{2}$ and optimal strategies $u_{1}^{*}\left(R_{1}\right)$ and $u_{1}^{*}\left(R_{2}\right)$ such that $u_{1}^{*}\left(R_{1}\right)>u_{1}^{*}\left(R_{2}\right)$. We will show that an individual with resource level *R*_2_ would achieve higher fitness by investing $u_{1}^{*}\left(R_{1}\right)$ in mating success than by investing $u_{1}^{*}\left(R_{2}\right)$, which contradicts the optimality of $u_{1}^{*}\left(R_{2}\right)$. Let $\Delta \;=u_{1}^{*}\;\;\left(R_{1}\right)-u_{1}^{*}\left(R_{2}\right)>0$. Because $R_{1}<R_{2}$ and $f_{\Delta }\left(u_{B}\right)$ is strictly decreasing, we have

(A4)
$$\begin{array}{ccc} \frac{x_{2}\left(R_{2}-u_{1}^{*}\;\left(R_{2}\right)\right)}{x_{2}\left(R_{2}-u_{1}^{*}\;\left(R_{1}\right)\right)} & & =f_{\Delta }\left(R_{2}-u_{1}^{*}\;\left(R_{1}\right)\right)<f_{\Delta }\left(R_{1}-u_{1}^{*}\left(R_{1}\right)\right)\\ & & =\;\frac{x_{2}\left(R_{1}-u_{1}^{*}\left(R_{2}\right)\right)}{x_{2}\left(R_{1}-u_{1}^{*}\left(R_{1}\right)\right)}. \end{array}$$

Further, if $u_{1}^{*}\left(R_{1}\right)$ is an optimal strategy for an individual with resource level *R*_1_, then it must yield fitness at least as high as any alternative strategy ${\hat{u}}_{1}$. In particular, when ${\hat{u}}_{1}=u_{1}^{*}\;\;\left(R_{2}\right)$, we must have

(A5)
$$\begin{array}{c} x_{1}\left(u_{1}^{*}\left(R_{1}\right)\right)x_{2}\left(R_{1}-u_{1}^{*}\left(R_{1}\right)\right)\geq x_{1}\left(u_{1}^{*}\left(R_{2}\right)\right)x_{2}\left(R_{1}-u_{1}^{*}\left(R_{2}\right)\right). \end{array}$$

Rearranging this equation yields

(A6)
$$\frac{x_{2}\left(R_{1}-u_{1}^{*}\;\left(R_{2}\right)\right)}{x_{2}\left(R_{1}-u_{1}^{*}\;\left(R_{1}\right)\right)}\leq \frac{x_{1}\left(u_{1}^{*}\;\left(R_{1}\right)\right)}{x_{1}\left(u_{1}^{*}\;\left(R_{2}\right)\right)}.$$

Lastly, combining the inequalities (A4) and (A6) and rearranging, we have

(A7)
$$\begin{array}{c} x_{1}\left(u_{1}^{*}\left(R_{2}\right)\right)x_{2}\left(R_{2}-u_{1}^{*}\left(R_{2}\right)\right)<x_{1}\left(u_{1}^{*}\left(R_{1}\right)\right)x_{2}\left(R_{2}-u_{1}^{*}\left(R_{1}\right)\right). \end{array}$$

In other words, the fitness of an individual with resource level *R*_2_ is higher when playing $u_{1}^{*}\left(R_{1}\right)$ than when playing $u_{1}^{*}\left(R_{2}\right)$. However, this contradicts the optimality of $u_{1}^{*}\left(R_{2}\right)$. Therefore, optimal investment in signaling is a nondecreasing function of an individual's resource level.

## Nonzero optimal investments at different resource levels can never be equal

Next, let us suppose that for some $R_{1}<R_{2}$ there exist optimal strategies $u_{1}^{*}\left(R_{1}\right)$ and $u_{1}^{*}\left(R_{2}\right)$ such that $u_{1}^{*}\;\;\left(R_{1}\right)=u_{1}^{*}\;\;\left(R_{2}\right)>0$. The corresponding optimal investments in viability are $R_{1}-u_{1}^{*}\left(R_{1}\right)$ and $R_{2}-u_{1}^{*}\left(R_{2}\right)$, respectively. From equations (6) and (7) in the main text, we have

(A8)
$$\begin{array}{ccc} S_{2}\left(R_{1}-u_{1}^{*}\left(R_{1}\right)\right) & & =S_{1}\;\;\left(u_{1}^{*}\left(R_{1}\right)\right)=S_{1}\;\;\;\left(u_{1}^{*}\left(R_{2}\right)\right)\\ & & =\;S_{2}\;\;\;\left(R_{2}-u_{1}^{*}\left(R_{2}\right)\right). \end{array}$$

However, because $R_{1}-u_{1}^{*}\left(R_{1}\right)<R_{2}-u_{1}^{*}\left(R_{2}\right)$, this contradicts the assumption that *S*_2_ is strictly decreasing. Therefore, if $R_{1}\neq R_{2}$, then nonzero optimal investments $u_{1}^{*}\left(R_{1}\right)$ and $u_{1}^{*}\left(R_{2}\right)$ can never be equal. Combined with the previous result, this means that if there exists a resource level $\hat{R}$ at which signaling investment is positive, then $u_{1}^{*}\left(R\right)$ is strictly increasing for all $R\geq \hat{R}$.

We can now combine the above results to prove the honest signaling theorem. Let $R^{*}\in \left[0,\infty \right]$ be the smallest resource level such that $R\leq R^{*}$ whenever $u_{1}\left(R\right)=0$ is optimal (i.e., $R^{*}=\sup\left\{R:u_{1}\left(R\right)=0\;\;\mathrm{is}\;\mathrm{optimal}\right\}$). Because $u_{1}^{*}\left(R\right)$ is nonnegative and nondecreasing, we must have $u_{1}^{*}\left(R\right)=0$ for all $R<R^{*}$. Further, because $u_{1}^{*}\left(R\right)$ is positive for all $R>R^{*}$, it is a strictly increasing function on the same interval.

## What determines *R* ^*^?

Here, we note some facts about the threshold resource level *R* ^*^ above which individuals signal:

(i) Individuals always signal (i.e., $R^{*}=0)$ if and only if $S_{1}\left(0\right)\geq S_{2}\left(0\right)$.
(ii) If $S_{1}\left(u_{1}\right)<S_{2}\left(u_{2}\right)$ for all *u*_1_ and *u*_2_, then individuals never signal (i.e., $R^{*}=\infty$).
(iii) If there exists a resource value $\hat{R}$ such that $S_{1}\left(0\right)>S_{2}\left(\hat{R}\right)$, then individuals signal for all $R\geq \hat{R}$ (i.e., $R^{*}\leq \hat{R}$). In particular, if the proportional marginal gains in viability tend to zero as investment increases (i.e., $S_{2}\left(u_{2}\right)\to 0$ as $u_{2}\to \infty )$, then there is a resource level above which individuals signal (i.e., $R^{*}<\infty$).

Propositions (ii) and (iii) are fairly straightforward. For (i), note that because *S*_2_ is strictly decreasing, $S_{1}\left(0\right)\geq S_{2}\left(0\right)$ implies that $S_{1}\left(0\right)>S_{2}\left(R\right)$ for all *R* > 0. Consequently, there can be no optimum of the form $u_{1}^{*}\;\left(R\right)=0$ and $u_{2}^{*}\;\left(R\right)=R$. Conversely, if $S_{1}\left(0\right)<S_{2}\left(0\right)$ then, by continuity, there is a value $R_{\delta }>0$ such that $S_{1}\left(u_{1}\right)<S_{2}\left(u_{2}\right)$ for all $u_{1}+u_{2}<R_{\delta }$. This implies that $u_{1}^{*}\;\left(R\right)=0$ is optimal for all $R\leq R_{\delta }$.

## When viability depends directly on signal size

In the main text, we assume that signal investment reduces viability indirectly, by consuming resources that would otherwise be invested in viability. However, signals might also reduce viability directly (e.g., by attracting predators). In this case, viability *x*_2_ is a function of both signal investment *u*_1_ and viability investment *u*_2_. Shifting resources from *u*_2_ to *u*_1_ reduces viability via two distinct mechanisms: reduced viability investment and increased signal size. We can then write fitness as

(A9)
$$W=x_{1}\;\left(u_{1}\right)x_{2}\;\left(u_{1},u_{2}\right)\;.$$

We write $S_{2}\left(u_{1},u_{2}\right)$ for the selection gradient that captures both of the viability effects associated with infinitesimal changes in *u*_2_:

(A10)
$$S_{2}\;\left(u_{1},u_{2}\right)=\frac{1}{x_{2}}\left(\frac{\partial x_{2}}{\partial u_{2}}-\frac{\partial x_{2}}{\partial u_{1}}\right).$$

Analogously to above, signaling is guaranteed to be honest if, for fixed *u*_1_, $S_{2}\left(u_{1},u_{2}\right)$ is a strictly decreasing function of *u*_2_:

(A11)
$$\begin{array}{c} \frac{\partial }{\partial u_{2}}S_{2}\;\left(u_{1},u_{2}\right)=\frac{1}{x_{2}}\left(\frac{\partial^{2}x_{2}}{\partial u_{2}^{2}}-\frac{\partial^{2}x_{2}}{\partial u_{1}\partial u_{2}}-\frac{\partial x_{2}}{\partial u_{2}}\;S_{2}\;\left(u_{1},u_{2}\right)\right)<0. \end{array}$$

In other words, for a fixed signal size, the proportional increase in viability when moving resources from *u*_1_ to *u*_2_ is a decreasing function of *u*_2_.

The proof is analogous to above. First, let us define

(A12)
$$f_{\Delta }\;\left(u_{1},u_{2}\right)=\frac{x_{2}\;\left(u_{1}-\Delta ,u_{2}+\Delta \right)}{x_{2}\;\left(u_{1},u_{2}\right)}.$$

We then have

(A13)
$$\begin{array}{c} \frac{\partial }{\partial u_{2}}f_{\Delta }\;\left(u_{1},u_{2}\right)=\frac{x_{2}\;\left(u_{1}-\Delta ,\;u_{2}+\Delta \right)}{x_{2}\;\left(u_{1},u_{2}\right)}\left(g_{u_{1},u_{2}}\left(\Delta \right)-g_{u_{1},u_{2}}\left(0\right)\right), \end{array}$$

where

(A14)
$$g_{u_{1},u_{2}}\;\left(\Delta \right)=\frac{x_{2}^{\left(0,1\right)}\left(u_{1}-\Delta ,u_{2}+\Delta \right)}{x_{2}\;\left(u_{1}-\Delta ,u_{2}+\Delta \right)}.$$

Here, we write *f* ^(0, 1)^ to represent the partial derivative of a binary function *f* with respect to its second argument, while holding the first argument fixed. Now note that

(A15)
$$\frac{\partial }{\partial \Delta }g_{u_{1},u_{2}}\;\left(\Delta \right)=S_{2}^{\left(1,0\right)}\;\;\left(u_{1}-\Delta ,u_{2}+\Delta \right)\;,$$

which is negative by assumption. Hence, $g_{u_{1},u_{2}}\left(\Delta \right)-g_{u_{1},u_{2}}\left(0\right)<0$ and so $\frac{\partial }{\partial u_{2}}f_{\Delta }\left(u_{1},u_{2}\right)<0$. In other words, the proportional increase in viability when reallocating Δ resources from *u*_1_ to *u*_2_ is a decreasing function of *u*_2_. The rest of the proof closely follows the one above.
